# Cerebrovascular pulsatility index is higher in chronic kidney disease

**DOI:** 10.14814/phy2.15561

**Published:** 2023-01-12

**Authors:** Ester S. Oh, Kaitlin A. Freeberg, Cortney N. Steele, Wei Wang, Heather Farmer‐Bailey, McKinley E. Coppock, Douglas R. Seals, Michel Chonchol, Matthew J. Rossman, Daniel H. Craighead, Kristen L. Nowak

**Affiliations:** ^1^ Division of Renal Diseases and Hypertension University of Colorado Anschutz Medical Campus Aurora Colorado USA; ^2^ Department of Integrative Physiology University of Colorado Boulder Boulder Colorado USA

**Keywords:** cerebrovascular, CKD, kidney, vascular

## Abstract

Patients with chronic kidney disease (CKD) are more likely to die of cardiovascular diseases, including cerebrovascular disease, than to progress to end‐stage kidney disease. Cerebrovascular dysfunction, characterized by reduced cerebrovascular reactivity, cerebral hypoperfusion, and increased pulsatile flow within the brain, precedes the onset of dementia and is linked to cognitive dysfunction. However, whether impaired cerebrovascular function is present in non‐dialysis dependent CKD is largely unknown. Using transcranial Doppler, we compared middle cerebral artery (MCA) blood velocity response to hypercapnia (normalized for blood pressure and end‐tidal CO_2_; a measure of cerebrovascular reactivity) and MCA pulsatility index (PI; a measure of cerebrovascular stiffness) in patients with stage 3–4 CKD vs. age‐matched healthy controls. We also administered the NIH cognitive toolbox (cognitive function), measured carotid‐femoral pulse‐wave velocity (PWV; aortic stiffness), and assessed ex vivo nitric oxide (NO) and reactive oxygen species (ROS) production from human brain endothelial cells incubated with serum obtained from study participants. MCA PI was higher in patients with CKD vs. controls; however, normalized MCA blood velocity response to hypercapnia did not differ between groups. Similar results were observed in a validation cohort of midlife and older adults divided by the median estimated glomerular filtration rate (eGFR). MCA PI was associated with greater large‐elastic artery stiffness (carotid‐femoral PWV), worse executive function (trails B time), lower eGFR, and higher ex vivo ROS production. These data suggest that impaired kidney function is associated with greater cerebrovascular stiffness, which may contribute to the known increased risk for cognitive impairment in patients with CKD.

## Graphical abstract

Middle cerebral artery (MCA) pulsatility index (PI) was higher, but cerebrovascular reactivity to hypercapnia was preserved in patients with non‐dialysis dependent CKD as compared to matched controls. MCA PI was positively associated with pulse‐wave velocity and ex vivo reactive oxygen species production from human brain endotheial cells incubated with serum from study participants and inversely associated with executive function. Further research is needed to confirm these results in larger cohorts and to delineate the mechanisms linking arterial stiffness, cerebrovascular function, and cognitive impairment in patients with CKD.

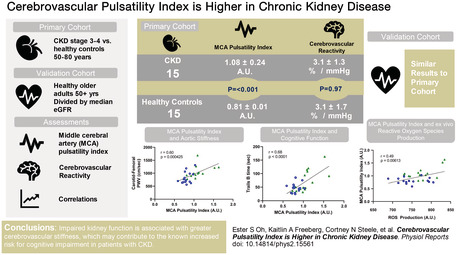

## 1 INTRODUCTION

Patients with chronic kidney disease (CKD) are more likely to die of cardiovascular diseases, including cerebrovascular disease, than to progress to end‐stage kidney disease (Sarnak et al., 2003). Impaired renal function is additionally associated with incident dementia (Seliger et al., 2004), incident cognitive impairment (Yaffe et al., 2008), and cognitive decline (Khatri et al., 2009). In recent years, there has been increased recognition of a kidney–brain axis, as both organs share anatomical and functional properties promoting vulnerability to vascular risk factors, such as high flow/low vascular resistance and local autoregulation (Lau et al., 2017; Lu et al., 2015).

Large‐artery vascular dysfunction is evident in patients with CKD, including impaired endothelium‐dependent dilation (Thambyrajah et al., 2000) and large‐elastic artery stiffening (Wang et al., 2005). Increased carotid‐femoral pulse‐wave velocity (PWV), the gold‐standard index of large‐elastic artery stiffness, is associated with worse performance on cognitive testing (Poels et al., 2007) and predicts cognitive decline, incident mild cognitive impairment, and incident dementia (Pase et al., 2016; Waldstein et al., 2008). Increased large‐artery stiffness promotes excessive flow pulsatility damage to the microcirculation, microvascular remodeling and microvascular parenchymal damage, and reduced cognitive performance (Cooper et al., 2016; Mitchell et al., 2011). Cerebrovascular dysfunction, characterized by reduced cerebrovascular reactivity, cerebral hypoperfusion, and increased pulsatile blood flow within the brain, precedes the clinical onset of dementia and has been linked to cognitive dysfunction (Nicolakakis & Hamel, 2011; Stone et al., 2015). However, whether impaired cerebrovascular function is present in non‐dialysis dependent CKD is largely unknown.

The primary goal of this study was to assess cerebrovascular function (change in mean blood velocity of the middle cerebral artery [ΔMV_MCA_] in response to vasodilatory hypercapnia [a measure of cerebrovascular reactivity] and MCA pulsatility index [PI; a measure of cerebrovascular stiffness]) in patients with stage 3–4 CKD as compared to a group of age‐matched healthy controls. Additionally, we assessed cognitive function, as well as large‐artery vascular function (carotid‐femoral PWV), circulating markers, and ex vivo nitric oxide (NO) and reactive oxygen species (ROS) production from human cerebrovascular endothelial cells treated with serum obtained from participants, as well as their association with measures of cerebrovascular function. We also sought to extend and confirm our findings to a validation cohort of midlife and older adults with varied kidney function. We hypothesized that participants with CKD would exhibit cerebrovascular dysfunction that associated with large‐artery stiffness, cognitive dysfunction, and differences in circulating markers, as well as ex vivo NO and ROS production from serum incubated brain endothelial cells.

## 2 METHODS

### 2.1 Study design and participants

#### 2.1.1 Primary cohort

This was a cross‐sectional study assessing cerebrovascular function in adults with stage 3–4 CKD and age‐matched healthy controls. The associations of cerebrovascular function with measures of cognitive function and large‐artery stiffness were also assessed. Participants with stage 3–4 CKD were recruited through a recruitment database and through a clinical relationship at the University of Colorado Anschutz Medical Campus CKD clinic. Healthy controls were recruited through university and community advertisement for comparison to participants with CKD. Enrollment occurred between April 2019 and March 2022. The study was conducted at the University of Colorado Anschutz Medical Campus Division of Renal Diseases and Hypertension Clinical Vascular Physiology Laboratory. Analysts were blinded to group (CKD or healthy control) in the assessment of all outcomes.

Inclusion criteria for both the CKD and control group were: 50–80 years of age (all women were postmenopausal), confirmation of a suitable temporal window for cerebrovascular assessments, and the ability to provide informed consent. Participants with CKD had an estimated glomerular filtration rate (eGFR) of 20–59 ml/min/1.73 m^2^ using the Chronic Kidney Disease Epidemiology Collaboration (CKD‐EPI) equation (Levey et al., 2009) and were required to have blood pressure controlled to <140/90 mm Hg and a stable antihypertensive regimen (if applicable) at the time of testing. Control participants had a CKD‐EPI eGFR ≥60 ml/min/1.73 m^2^, were free from hypertension (defined as current treatment or blood pressure ≥ 140/90 mmHg), as well as kidney disease (i.e., proteinuria), cardiovascular disease, diabetes, and other chronic diseases, as assessed by self‐report, medical history and physical exam (including a resting 12‐lead electrocardiogram), and screening laboratories. Additional exclusion criteria in both groups were dementia, history of stroke, diagnosis of a major psychiatric disorder (e.g., psychosis, schizophrenia, mania, bipolar disorder), medications likely to affect central nervous system functions (e.g., long‐active benzodiazepines), current diagnosis and treatment of major depression (based on DSM‐IV‐TR criteria for Major Depressive Episode), alcohol or other substance abuse (self‐report or undergoing treatment), significant sensory or motor deficits that would interfere with cognitive testing, active infection or antibiotic therapy, hospitalization in the last month, immunosuppressive therapy within the last year, body mass index (BMI) >40 kg/m^2^, current tobacco or nicotine use or history of use in the last 12 months, oral hormone therapy, cannabis use within 2 weeks prior to testing, and antioxidant and/or omega‐3 fatty acid use within 2 weeks prior to testing.

#### 2.1.2 Validation cohort

To confirm our findings in the primary cohort, we assessed a validation cohort of participants in research studies at the University of Colorado Boulder Integrative Physiology of Aging Laboratory. The validation cohort was comprised of all individuals aged 50 years and older studied in the laboratory between June 2021 (when the current cerebrovascular measurements were established) and May 2022 (when data analysis for the present manuscript was performed). Participants were recruited through a recruitment database and community advertisement. These individuals were generally healthy midlife and older adults (women were postmenopausal) and free from overt cardiovascular diseases and dementia, with a BMI <40 mg/m^2^ and no recent changes in medications or health status. Participants in this validation cohort were divided in half based upon the median CKD‐EPI eGFR (86 ml/min/1.73 m^2^). While a slightly higher eGFR than the primary cohort, inclusion of the goal of the validation cohort provided some initial evidence that changes in cerebrovascular function occur early in the process of kidney function decline.

### 2.2 Procedures

#### 2.2.1 Screening measures

A metabolic panel, complete blood count, and lipid panel were performed. Demographic data and medical history were collected by self‐report. Arterial blood pressure was measured in triplicate while seated at rest using an automated oscillometric machine.

#### 2.2.2 Cerebrovascular measurements

All measurements were made following standard recommendations including an overnight fast (Harris et al., 2010). Cerebrovascular reactivity was measured by assessing ΔMV_MCA_ in response to a vasodilatory hypercapnic challenge (i.e., via CO_2_ breathing) using transcranial Doppler (TCD; Lucid M1 System, NovaSignal) as described previously (Edwards et al., 2004; Zuj et al., 2012). MCA blood velocity (MCAv) was continually assessed using a 2‐MHz TCD ultrasound probe positioned at the temporal window and held in place by an adjustable headband. The temporal window is the thinnest portion of the temporal bone and thus offers an ideal exposure for Doppler ultrasonography of the circle of Willis arteries, including the MCA (Purkayastha & Sorond, 2012). Following baseline recording (room air), participants breathed room air mixed with 5% CO_2_ (balanced nitrogen) to induce mild hypercapnia. For the primary cohort, each condition was recorded for 5 min in the seated position to achieve a steady‐state velocity with paced breathing using a metronome. For the validation cohort, assessments were made in the supine position, the duration of hypercapnia was 4 min, and breathing was self‐paced. MV_MCA_ was determined for each condition by calculating the average MCAv over each cardiac cycle for the last minute of each condition. Percent ΔMV_MCA_ was calculated as: [(hypercapnic MV_MCA_−normocapnic MV_MCA_)/(normocapnic MV_MCA_)] × 100 and used to compare cerebrovascular reactivity between groups.

Breath‐by‐breath end‐tidal partial pressure of CO2 (ETCO2) was continuously monitored (Vacumed) and was used to normalize the MV_MCA_ response to changes in arterial blood gas (Purkayastha & Sorond, 2012). The assessment of ETCO2 is noninvasive and correlates strongly with (invasive) serial blood measures of arterial blood gas (Purkayastha & Sorond, 2012). For the primary cohort, brachial artery blood pressure was measured using an automated cuff during minute two of each condition to normalize the MV_MCA_ response for change in blood pressure; for the validation cohort, beat‐by‐beat blood pressure (FinometerPro) calibrated to brachial artery blood pressure was used. Relative cerebrovascular reactivity was calculated as %ΔMV_MCA_/(hypercapnia ETCO_2_–resting ETCO_2_). Vascular conductance was calculated as MV_MCA_/mean arterial pressure for each condition. Percent Δ in MCA vascular conductance (VC_MCA_) was calculated as [(hypercapnic VC_MCA_–normocapnic VC_MCA_)/(normocapnic VC_MCA_)] × 100. Relative VC_MCA_ reactivity was calculated as % ΔVC_MCA_/(hypercapnia ETCO_2_ – resting ETCO_2_).

Pulsatile cerebrovascular velocity was determined using the Gosling PI, calculated as PI = (MCAv_(systolic)_ − MCAv_(diastolic)_)/MV_MCA_ as described previously (Brar et al., 2016). PI is positively associated with arterial stiffness (Mitchell et al., 2011) and implies an impaired ability of the cerebral arteries to buffer the large increases in pressure delivered to the brain via the heart, through the carotid arteries.

In the primary cohort, cerebral blood flow (CBF) was assessed in the semi‐seated position (45‐degree angle at the hips) using extracranial high‐resolution duplex ultrasound (Xario 200, Canon Medical Systems) to capture both diameter and time‐averaged mean blood velocity (MV) of the right internal carotid artery (ICA) and vertebral artery (VA) (i.e., the two primary arteries that supply the brain). ICA and VA diameter were measured over 30 s each during the diastolic phase of the cardiac cycle and averaged. CBF was calculated for each artery as follows: CBF = MFV × *π*(diameter/2)^2^, as described previously (Fraser et al., 2015; Robertson et al., 2010). The sum of CBF calculated for the right artery was multiplied by two for a bilateral estimate of total CBF (Robertson et al., 2010).

#### 2.2.3 Arterial stiffness

Carotid‐femoral PWV was measured as described in detail previously (Jablonski et al., 2014; Nowak et al., 2017, 2020). Briefly, a transcutaneous custom tonometer (Noninvasive Hemodynamics Workstation [NIHem], Cardiovascular Engineering Inc.) was positioned at the carotid, radial and femoral arteries to noninvasively assess carotid‐femoral PWV and carotid‐radial PWV (an index of peripheral stiffness).

#### 2.2.4 Cognitive function

The NIH Toolbox for the Assessment of Neurological and Behavioral Function was initiated by the NIH Blueprint for Neuroscience Research to develop state‐of‐the‐art measurement tools for the collection of cognitive data, to meet the need for a standard set of measures that can be used as a “common currency” across diverse study designs and settings (Gershon et al., 2013). A higher score indicates better cognitive performance. The NIH Cognitive Toolbox is a validated multidimensional assessment of cognitive function designed to assess a wide range of cognitive subdomains in a brief amount of time (Weintraub et al., 2014). Additionally, we administered the Trail Making Test (parts A and B) as indices of processing speed and executive function, respectively, with a shorter time to complete the test indicating better performance (Kurella et al., 2004).

#### 2.2.5 Circulating markers

Samples to analyze circulating markers were available from the primary cohort. Brain‐derived neurotrophic factor (BDNF; a key neurotrophic factor and marker of blood–brain barrier disruption), interleukin‐1β (IL‐1β), IL‐6, tumor necrosis factor‐α (TNF‐α) (pro‐inflammatory cytokines), and monocyte chemoattractant protein (MCP‐1; chemokine regulating migration/infiltration of monocytes/macrophages) were measured using fasting plasma samples (1:2 dilution, Customized U‐Plex Metabolic Group 1, Meso Scale Discovery, Cat# K15052K‐1, Lot# 356950). Soluble CD14 (sCD14; marker of gut‐blood barrier permeability) was measured by ELISA using fasting serum samples (1:200 dilution; R&D Systems, Cat# DC140, Lot# P319505). Enolase 2 (neuron‐specific, marker of blood–brain barrier disruption) was measured in fasting serum samples by ELISA (no dilution, R&D Systems, Cat# DENL20, lot P316287). Superoxide dismutase (SOD; antioxidant enzyme) activity was measured in fasting serum samples (1:4 dilution, Invitrogen, Cat# EIASODC, lot # 22SD001D).

#### 2.2.6 Serum incubation model in cerebrovascular endothelial cell culture

To determine whether circulating factors may contribute to differences in cerebrovascular endothelial function between groups, we performed ex vivo experiments whereby human brain endothelial cells (HBECs) were treated with serum from the primary cohort. HBECs (ATCC Cat# CRL‐3245, RRID:CVCL_4D10) were plated in 96‐well culture plates and incubated under standard conditions (37°C, 5% CO_2_) for 2 h in basal media supplemented with 10% subject serum. After serum exposure, cells were co‐incubated with the fluorescent probes CellROX Deep Red (Thermo Fisher Scientific, Cat# C10422) to detect ROS production and DAR‐4 M‐AM (Sigma‐Aldrich, Cat# 251765) to detect NO production (Craighead et al., 2021; Rossman et al., 2021). Cells were imaged before and 6 minutes after addition of 100 μM acetylcholine to stimulate NO production. Analysis was done with Celleste Image Analysis Software (Cellesta, v5, RRID:SCR_022692). ROS production was normalized to signal area and NO production was quantified as the fold change of post vs. pre‐acetylcholine stimulation.

### 2.3 Statistical analyses and power calculations

Differences in variables between groups were assessed using independent samples *t*‐tests, chi‐squared tests, or Fisher's exact tests. Non‐normally distributed variables were log‐transformed prior to analysis (enolase 2, BDNF, IL‐1β, IL‐6, TNF‐α, and MCP‐1). All data are reported as means ± SD or medians (interquartile range). ANCOVA was used to adjust for covariates when comparing outcomes between groups. Pearson's bivariate correlations and multiple linear regression were used to evaluate associations between variables and sex differences (group × sex interaction). Analyses were performed using SPSS 27 and statistical significance was set at *p* < 0.05 with a two‐sided alpha. As the data were considered hypothesis‐generating, adjustment was not made for multiple comparisons.

An effect size was estimated for a primary outcome of cerebrovascular reactivity based on a previous cross‐sectional study comparing patients with anemia secondary to kidney failure and healthy controls (Kuwabara et al., 2002). Calculations were performed using G‐power 3.1 using an independent samples *t*‐test. Based on a value of 5.26 ± 0.82 in the control group and 2.6 ± 1.18 in the kidney failure group, with an effect size of 2.20, 10 participants would be needed per group with 80% power and an α‐level of 0.05. As we anticipated that the effect size may not be as large for the difference between patients with non‐dialysis dependent CKD and controls, we increased the number of participants needed by 50% (to 15 per group).

## 3 RESULTS

### 3.1 Primary cohort

#### 3.1.1 Demographic and clinical characteristics

In the primary cohort, 20 participants with stage 3–4 CKD were assessed for eligibility and five were excluded from enrollment (5 did not meet inclusion/exclusion criteria, 0 declined to participate), for a total CKD cohort of 15. Twenty‐one control participants were assessed for eligibility and six were excluded from enrollment (5 did not meet inclusion/exclusion criteria, 1 declined to participate), for a total control cohort of 15. Healthy control participants had a lower BMI (Table 1). The majority of participants with CKD had hypertension and 40% had diabetes mellitus, which were exclusion criteria for healthy controls. Participants with CKD (eGFR of 39 ± 13 ml/min/1.73 m^2^) were treated with antihypertensive agents not used by controls and were more likely to be treated with a statin.

**TABLE 1:** Demographics and clinical characteristics of chronic kidney disease and control participants

Variable	CKD (*n* = 15)	Control (*n* = 15)	*p*‐value
Age (years)	69 ± 7	65 ± 7	0.118
Sex, *n* (%) Male	11 (73)	9 (60)	0.700
Race/Ethnicity, % Non‐Hispanic White	9 (60)	13 (87)	0.215
Education			
Some college	5 (33%)	3 (20%)	0.219
College graduate	6 (40%)	3 (20%)	
Advanced degree	4 (27%)	9 (60%)	
BMI (kg/m^2^)	31.7 ± 3.6	26.3 ± 4.3	0.000818
Systolic BP (mm Hg)	122 ± 13	116 ± 10	0.157
Diastolic BP (mm Hg)	67 ± 8	71 ± 6	0.148
eGFR, ml/min/1.73 m^2^	39 ± 13	84 ± 12	<0.0001
Glucose (mg/dl)	117 ± 49	96 ± 11	0.127
LDL cholesterol (mg/dl)	93 ± 46	104 ± 25	0.421
HDL cholesterol (mg/dl)	52 ± 28	64 ± 22	0.227
Total cholesterol (mg/dl)	169 ± 59	185 ± 38	0.379
Hemoglobin (g/dl)	14.2 ± 1.8	15.0 ± 1.1	0.176
Hematocrit (%)	42.8 ± 5.0	44.7 ± 2.8	0.216
24‐hr urine volume (ml)	2084 ± 887	2296 ± 1212	0.598
24‐hr urinary sodium (mmol/day)	140 ± 60	139 ± 36	0.948
24‐hr urinary potassium (mmol/day)	52 ± 16	80 ± 25	0.00178
24‐hr urinary creatinine (mg/day)	1312 ± 318	1419 ± 426	0.227
24‐hr urinary protein (mg/day)	133 (88, 493)	104 (84, 205)	0.426
Hypertension (%)	12 (80)	0	<0.0001
Diabetes (%)	6 (40)	0	0.0169
CAD or CHF (%)	3 (20)	0	0.224
ACEi/ARB (%)	9 (60)	0	0.00700
Diuretic (%)	8 (53)	0	0.00220
Beta Blocker (%)	4 (27)	0	0.100
Calcium channel blockers (%)	7 (47)	0	0.00632
Statin (%)	10 (67)	2 (13)	0.00778
Proton pump inhibitor (%)	5 (33)	0	0.0421
Antianxiety medication (%)	3 (20)	1 (7)	0.598
Thyroid medication (%)	3 (20)	3 (20)	1.000

*Note*: Data are mean ± SD or *n* (%). Statistical comparisons are by a chi‐square or Fisher's exact tests for categorical data and an independent sample t‐test for continuous variables.

Abbreviations: ACEi, angiotensin converting enzyme inhibitor; ARB, angiotensin receptor blocker.; BMI, body mass index; BP, blood pressure (seated position); CAD, coronary artery disease; CHF, congestive heart failure; CKD, chronic kidney disease; eGFR; estimated glomerular filtration rate (by the Chronic Kidney Disease Epidemiology Collaboration equation); HDL, high‐density lipoprotein; LDL, low‐density lipoprotein.

#### 3.1.2 Cerebrovascular measurements

**FIGURE 1:**
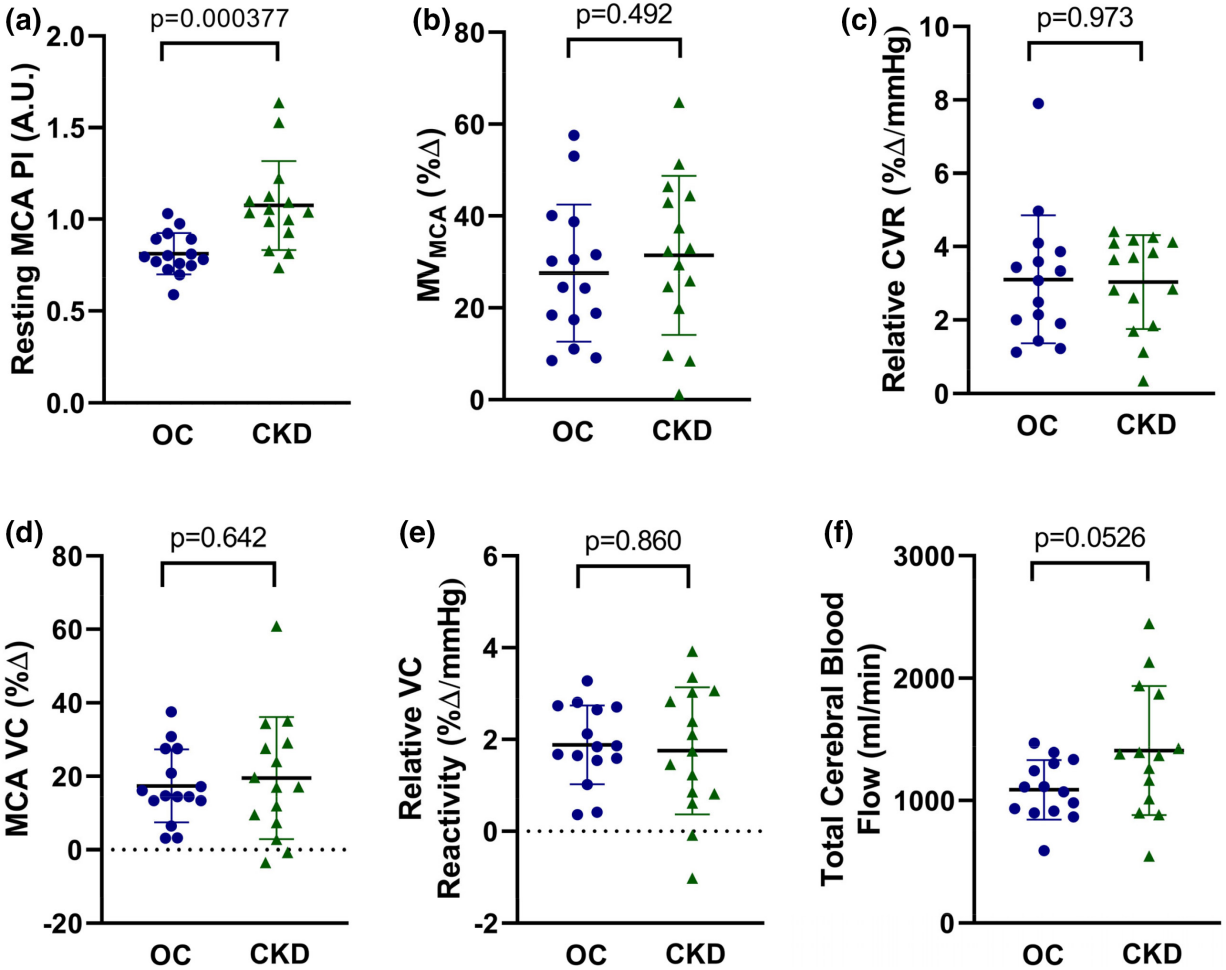
Resting pulsatility index (PI) of the middle cerebral artery (MCA), calculated as (MCA velocity systole—MCA velocity diastole)/(MCA velocity mean) (panel a). Percent change in mean flow velocity of the MCA (MV_MCA_) in response to hypercapnia in older control (OC; blue circles) and chronic kidney disease (CKD; green triangles) participants (panel b). Relative cerebrovascular reactivity (CVR), calculated as percent change in MV_MCA_ normalized to absolute change in end‐tidal CO_2_ in response to hypercapnia (panel c). Percent change in vascular conductance (VC; MV_MCA_/mean arterial pressure) (panel d). Percent change in relative VC, calculated as percent change in VC normalized to absolute change in end‐tidal CO_2_ in response to hypercapnia (panel e). Total cerebral blood flow, calculated as right internal carotid blood flow × 2 + right vertebral artery blood flow × 2 (panel f). Horizontal lines represent mean, vertical lines represent s.d., and circles/triangles represent individual participants. Comparisons between groups were evaluated by an independent sample *t*‐test.

Participants with CKD had a 32% higher MCA PI as compared to healthy controls (CKD: 1.08 ± 0.24 AU; control: 0.81 ± 0.011 AU; *p* = 0.000377; Figure 1a). This group difference remained significant when co‐varying for statin usage (*p* = 0.0382), or BMI (*p* = 0.0160), but not for hypertension (*p* = 0.250). However, resting MV_MCA_ did not differ between groups (CKD: 46.0 ± 5.6 cm/s; control: 48.1 ± 13.2 cm/s; *p* = 0.576). There was no difference in cerebrovascular reactivity between participants with CKD and healthy controls, whether expressed as %ΔMV_MCA_ (CKD: 27.6 ± 14.9%Δ; control: 31.8 ± 18.1%Δ; *p* = 0.492; b), relative cerebrovascular reactivity (CKD: 3.1 ± 1.3%Δ/mmHg; control: 3.1 ± 1.7%Δ/mmHg; *p* = 0.973; c), %Δ VC_MCA_ (CKD: 19.9 ± 17.6%Δ; control: 17.4 ± 9.9%Δ; *p* = 0.642; d), or relative MCA VC reactivity (CKD: 1.8 ± 1.5%Δ/mmHg; control: 1.9 ± 0.86%Δ/mmHg; *p* = 0.860; e). Peak ETCO_2_ also did not differ between groups (CKD: 41.1 ± 2.3 mmHg; control: 43.7 ± 5.6 mmHg; *p* = 0.115). Total CBF was not significantly different in the CKD group as compared to the control group (CKD: 1409 ± 527 ml/min; control: 1088 ± 243 ml/min; *p* = 0.0526; f).

**FIGURE 2:**
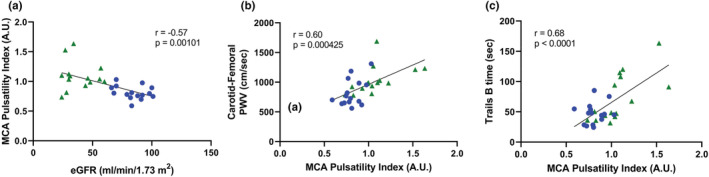
Association of estimated glomerular filtration rate (eGFR) with middle cerebral artery (MCA) pulsatility index (panel a), middle cerebral artery pulsatility index with carotid‐femoral pulse‐wave velocity (PWV) (panel b), and MCA pulsatility index with trails B (trail making test part B) time (panel c), with older control in blue circles and chronic kidney disease in green triangles using Pearson's bivariate correlations.

There was a significant group × sex interaction (*p* = 0.00450) for PI. The group difference in MCA PI was significant in males (CKD: 1.12 ± 0.26 A.U. [*n* = 11]; control: 0.81 ± 0.08 A.U. [*n* = 9]; *p* = 0.00303), but not in females (CKD: 0.96 ± 0.16 A.U. [*n* = 4]; control: 0.81 ± 0.16 A.U. [*n* = 6]; *p* = 0.205), albeit sample size was greater in males. Estimated GFR was inversely correlated with MCA PI (*r* = −0.57, *p* = 0.00101; Figure 2a). This association persisted after correction for age and sex (*β* = −0.42, *p* = 0.00793).

#### 3.1.3 Arterial stiffness

Consistent with previous reports, carotid‐femoral PWV was greater in participants with CKD as compared to healthy controls (CKD: 1031 ± 237 cm/s; control: 843 ± 221 cm/s; *p* = 0.0330). Carotid‐femoral PWV was positively correlated with MCA PI (*r* = 0.60, *p* = 0.000425; Figure 2b). This association persisted after correction for age, sex, and eGFR (*β* = 0.39, *p* = 0.0137).

#### 3.1.4 Cognitive function

Participants with CKD had a slower time on the trail making part B test but did not significantly differ in NIH toolbox cognitive test scores as compared to healthy controls (Table 2). MCA PI was positively correlated with trail making part B test time (*r* = 0.68, *p* < 0.0001; Figure 2c). This association persisted after adjustment for age, sex, eGFR, and education (*β* = 0.73, *p* = 0.00106). Hemoglobin was correlated with uncorrected processing speed (*r* = 0.43, *p* = 0.0217), which also persisted after correction for age, sex, eGFR, and education (*β* = 0.47, *p* = 0.0400).

**TABLE 2:** Cognitive function in chronic kidney disease and control participants

Variable	CKD (*n* = 15)	Control (*n* = 15)	*p*‐value
Trails A (s)	27.9 ± 9.5	22.4 ± 5.6	0.0672
Trails B (s)	73.0 ± 39.8	47.7 ± 16.6	0.0354
Picture vocabulary, score	112.5 ± 10.0	117.6 ± 8.8	0.170
Flanker, score	99.3 ± 7.3	95.6 ± 7.9	0.190
List sort, score	97.3 ± 13.8	102.0 ± 9.0	0.284
Dimensional card sort, score	102.7 ± 8.9	104.1 ± 6.9	0.635
Processing speed, score	91.1 ± 16.3	95.3 ± 12.7	0.445
Sequence memory, score	97.9 ± 12.3	94.6 ± 12.6	0.479
Reading recognition, score	109.5 ± 5.8	110.7 ± 8.1	0.654
Fluid composite, score	95.4 ± 11.4	96.5 ± 7.4	0.750
Crystallized composite, score	111.3 ± 8.3	114.8 ± 8.3	0.285
Total composite, score	103.5 ± 10.6	106.4 ± 6.9	0.403

*Note*: Data are mean ± SD. Statistical comparisons are by an independent sample *t*‐test. A shorter time to complete the test indicates better performance. The NIH toolbox scores are uncorrected scores.

Abbreviations: CKD, chronic kidney disease; Trails A, trail making test part A; trails B, trail making test part B.

#### 3.1.5 Circulating markers

Soluble CD14 and SOD levels were higher in CKD as compared to control participants (Table 3). There were no significant group differences in enolase 2 (*p* = 0.956), BDNF (*p* = 0.171), IL‐1β (*p* = 0.980), IL‐6 (*p* = 0.0561), MCP‐1 (*p* = 0.135), or TNF‐α (*p* = 0.0757). Greater (log‐transformed) sCD14, adjusted for age, sex, and eGFR was associated with higher MCA PI (*β* = 0.54, *p* = 0.00679).

**TABLE 3:** Circulating markers in chronic kidney disease and control participants

Variable	CKD (*n* = 15)	Control (*n* = 15)	*p*‐value
sCD14 (ng/ml)	2.1 ± 0.48	1.7 ± 0.22	0.00470
Enolase 2 (ng/ml)	3.7 (2.7, 4.0)	3.4 (3.0, 4.1)	0.956
BDNF (pg/ml)	662 ± 423	476 ± 291	0.171
IL‐1β (pg/ml)	5.2 (3.2, 8.1)	4.2 (3.6, 8.0)	0.980
IL‐6 (pg/ml)	4.5 (2.9, 6.7)	2.8 (1.2, 4.3)	0.0561
MCP‐1 (pg/ml)	96.7 (78.7, 114.1)	79.9 (73.0, 88.6)	0.134
TNF‐α (pg/ml)	1.8 (1.3, 2.4)	1.0 (0.62, 1.9)	0.0757
SOD (U/ml)	1.24 ± 0.0.46	0.91 ± 0.10	0.0147

*Note*: Data are mean ± S.D or median (IQR). Statistical comparisons are by an independent sample *t*‐test with log‐transformation before analysis for skewed variables.

Abbreviations: BDNF, bone‐derived neurotrophic factor; CKD, chronic kidney disease; IL‐1β, interleukin‐1β; IL‐6, interleukin‐6; MCP1, monocyte chemoattractant protein‐1; sCD14, soluble CD14; SOD, superoxide dismutase.; TNF‐α, tumor necrosis factor‐α.

#### 3.1.6 Serum incubation model

**FIGURE 3:**
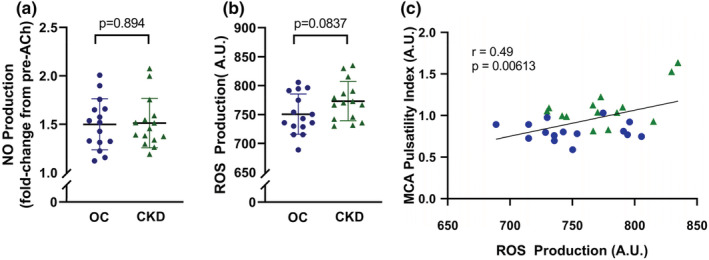
Production of nitric oxide (NO, in response to acetylcholine [ACh] panel a) and reactive oxygen species (ROS, panel b) after 2 h of exposure to serum from old control (OC) participants (blue) or participants with chronic kidney disease (CKD, green) in human brain endothelial cells (HBEC‐5is, ATCC). Horizontal lines represent mean, vertical lines represent s.d., and circles/triangles represent individual participants. Comparisons between groups were evaluated by an independent sample *t*‐test. ROS production is normalized to signal area. The correlation between ROS production and middle cerebral artery (MCA) resting pulsatility index (Pearson's bivariate correlation) is shown in (panel c)

HBEC production of NO (fold change from pre‐acetylcholine; Figure 3a) and ROS bioactivity (AU; Figure 3b) did not differ following exposure to serum from participants with CKD as compared to healthy controls (NO: control: 1.51 ± 0.26, CKD: 1.50 ± 0.27, *p* = 0.894; ROS: control: 750.4 ± 34.8, CKD: 773.0 ± 34.0, *p* = 0.0837). However, greater ROS production was positively correlated with higher MCA PI (*r* = 0.49, *p* = 0.00613; Figure 3c), which was attenuated after adjustment for age (*β* = 0.54, *p* = 0.191).

### 3.2 Validation cohort

**FIGURE 4:**
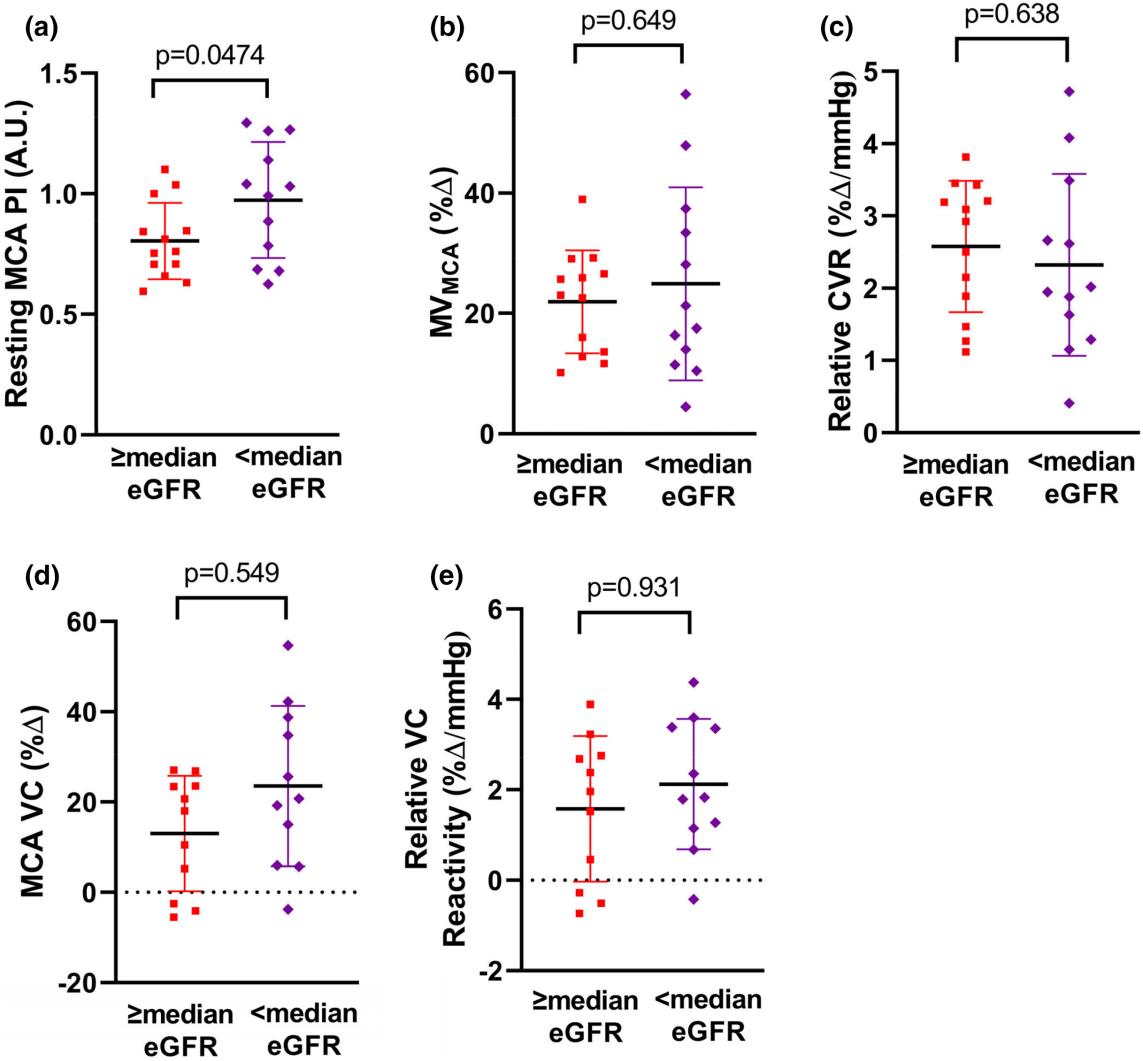
Resting pulsatility index (PI) of the middle cerebral artery (MCA), calculated as (MCA velocity systole—MCA velocity diastole)/(MCA velocity mean) (panel a). Percent change in mean velocity of the MCA (MV_MCA_) in response to hypercapnia in participants with a CKD‐EPI eGFR ≥median (red squares) and < median (purple diamonds) (panel b). Relative cerebrovascular reactivity (CVR), calculated as percent change in MV_MCA_ normalized to absolute change in end‐tidal CO_2_ in response to hypercapnia (panel c). Percent change in vascular conductance (VC; MV_MCA_/mean arterial pressure) (panel d). Percent change in relative VC, calculated as percent change in VC normalized to absolute change in end‐tidal CO_2_ in response to hypercapnia (panel e). Horizontal lines represent mean, vertical lines represent s.d., and squares/diamonds represent individual participants. Comparisons between groups were evaluated by an independent sample *t*‐test. *N* = 2 with eGFR ≥ median and *n* = 1 with eGFR <median missing in panel (c) and (d) due to missing mean arterial pressure.

**FIGURE 5:**
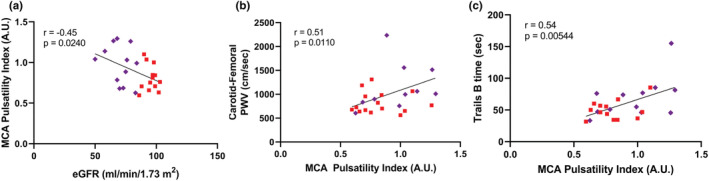
Association of estimated glomerular filtration rate (eGFR) with middle cerebral artery (MCA) pulsatility index (panel a), MCA pulsatility index with carotid‐femoral pulse‐wave velocity (PWV) (panel b), and MCA pulsatility index with trails B (trail making test part B) time (panel c), with participants with a CKD‐EPI eGFR ≥ median in red squares and < median in purple diamonds. Comparisons between groups were evaluated by an independent sample *t*‐test.

Participant characteristics of the validation cohort are provided in Table 4. These participants were divided into two groups by the median CKD‐EPI eGFR (86 ml/min/1.73 m^2^) but did not significantly differ in other characteristics or in cognitive function (Table 5). Similar to the primary cohort, individuals with a lower eGFR had a significantly higher resting MCA PI, but no difference in %ΔMV_MCA_, relative cerebrovascular reactivity, %Δ VC_MCA_, or relative MCA VC reactivity (Figure 4). Resting MV_MCA_ did not differ between groups (<median: 44.9 ± 17.8 cm/s; ≥median: 46.5 ± 13.8 cm/s *p* = 0.801). Also similar to the primary cohort, eGFR was inversely associated with MCA PI, and MCA PI was positively associated with carotid‐femoral PWV and trail making time part B (Figure 5). There was no significant group ×sex interaction for MCA PI (*p* = 0.600).

**TABLE 4:** Demographics and clinical characteristics of validation cohort

Variable	All participants (*n* = 25)	eGFR < median (*n* = 12)	eGFR ≥ median (*n* = 13)	*p*‐value (≥ vs. <median)
Age (years)	68 ± 9	72 ± 10	65 ± 8	0.0621
Sex, *n* (%) Male	8 (32)	3 (25)	5 (38)	0.673
Race/Ethnicity, *n* (%) Non‐Hispanic White	24 (96)	11 (92)	13 (100)	0.480
Education(%)				
Some college or HS degree	3 (12)	1 (8)	2 (15)	1.000
College graduate	9 (36)	5 (42)	4 (31)	
Advanced degree	13 (52)	6 (50)	7 (54)	
BMI (kg/m^2^)	25.4 ± 3.7	25.7 ± 3.3	25.1 ± 4.2	0.712
Systolic BP (mm Hg)	124 ± 12	124 ± 13	125 ± 12	0.890
Diastolic BP (mm Hg)	76 ± 10	74 ± 7	77 ± 13	0.591
eGFR, ml/min/1.73m^2^	84 ± 15	71 ± 10	95 ± 5	<0.0001
Glucose (mg/dl)	94 ± 11	93 ± 7	94 ± 14	0.811
LDL cholesterol (mg/dl)	102 ± 29	94 ± 25	109 ± 31	0.863
HDL cholesterol (mg/dl)	60 ± 15	61 ± 16	60 ± 14	0.201
Total cholesterol (mg/dl)	181 ± 40	173 ± 36	189 ± 43	0.322
Hemoglobin (g/dl)	14.1 ± 1.0	13.7 ± 0.82	14.4 ± 1.0	0.061
Hematocrit (%)	42.7 ± 2.5	42.1 ± 2.4	43.3 ± 2.4	0.204
Hypertension, *n* (%)	15 (60)	7 (58)	8 (62)	1.000
Diabetes, *n* (%)	1 (4)	0 (0)	1 (8)	1.000
CAD or CHF, *n* (%)	0	0	0	1.000
ACEi/ARB, *n* (%)	7 (28)	2 (17)	5 (38)	0.378
Diuretic, *n* (%)	2 (8)	2 (17)	0 (0)	0.220
Beta blocker, *n* (%)	0	0	0	1.000
Calcium channel blockers, *n* (%)	3 (12)	1 (8)	2 (15)	1.000
Statin, *n* (%)	8 (32)	6 (50)	2 (15)	0.0968
Proton pump inhibitor, *n* (%)	2 (4)	1 (8)	1 (8)	1.000
Antianxiety medication, *n* (%)	2 (4)	1 (8)	1 (8)	1.000
Antidepressant medication, *n* (%)	2 (4)	2 (17)	0 (0)	0.220
Thyroid medication, *n* (%)	4 (16)	3 (25)	1 (8)	0.322

*Note*: Data are mean ± S.D. or *n* (%). Statistical comparisons are by a chi‐squared or Fisher's exact tests for categorical data and an independent sample t‐test for continuous variables.

Abbreviations: ACEi, angiotensin converting enzyme inhibitor; ARB, angiotensin receptor blocker; BMI, body mass index; BP, blood pressure (seated position); CAD, coronary artery disease; CHF, congestive heart failure; eGFR; estimated glomerular filtration rate (median eGFR by the Chronic Kidney Disease Epidemiology Collaboration equation is 86 mL/min/1.73m^2^); HDL, high‐density lipoprotein; LDL, low‐density lipoprotein.

**TABLE 5:** Cognitive function in validation cohort

Variable	eGFR < median (*n* = 12)	eGFR ≥ median (*n* = 13)	*p*‐value
Trails A (s)	22.2 ± 7.2	24.7 ± 10.5	0.501
Trails B (s)	49.9 ± 15.2	68.9 ± 32.1	0.0679
Picture vocabulary, score	120.3 ± 6.5	120.3 ± 8.1	0.993
Flanker, score	99.8 ± 8.3	98.6 ± 6.4	0.693
List sort, score	104.2 ± 12.7	98.0 ± 6.4	0.140
Dimensional card sort, score	107.2 ± 6.3	103.8 ± 7.5	0.229
Processing speed, score	105.0 ± 17.2	102.8 ± 11.7	0.708
Sequence memory, score	101.7 ± 13.5	100.3 ± 15.8	0.808
Reading recognition, score	115.0 ± 4.6	116.7 ± 4.9	0.390
Fluid composite, score	103.7 ± 10.5	99.8 ± 9.2	0.340
Crystallized composite, score	118.4 ± 5.3	119.4 ± 5.6	0.643
Total composite, score	112.5 ± 7.6	110.8 ± 7.2	0.553

*Note*: Data are mean ± SD. Group differences evaluated by an independent sample *t*‐test. eGFR, estimated glomerular filtration rate (median eGFR by the Chronic Kidney Disease Epidemiology Collaboration equation is 86 ml/min/1.73 m^2^); Trails A, trail making test part A; trails B, trail making test part B. A shorter time to complete the test indicates better performance. The NIH toolbox scores are uncorrected scores.

## 4 DISCUSSION

In this cross‐sectional study, MCA PI was significantly greater in patients with non‐dialysis dependent CKD as compared to healthy controls, and MCA PI was associated with greater large‐elastic artery stiffness (CFPWV), worse executive function (trails B time), lower eGFR, and greater ex vivo ROS production. In contrast, MCA cerebrovascular reactivity and total CBF did not differ between groups. Importantly, similar results were observed in a validation cohort of midlife and older adults divided by the median eGFR.

Patients with CKD exhibited significantly greater MCA PI, an index of cerebrovascular stiffness, as compared to healthy controls, consistent with a recent observation in patients with lacunar infarction with and without CKD (Lee et al., 2021). In the current study, higher MCA PI was independently associated with reduced kidney function (eGFR), greater large‐artery stiffness (carotid‐femoral PWV), greater ex vivo ROS production in serum incubation cell culture studies, as well as reduced cognitive function (slower trail making part B time). With increased aortic stiffness, blood flow is delivered with excessive pulsatile energy, promoting end‐organ damage, including in the cerebral microcirculation (Mitchell et al., 2011). Increased large‐artery stiffness is associated with impaired cerebral microvascular function, brain structural changes, and cognitive impairment in non‐CKD cohorts (Al Hazzouri & Yaffe, 2014; Cooper et al., 2016; Mitchell et al., 2011). Related, MCA PI is significantly greater in patients with hypertension who also have cognitive impairment (Harris et al., 2018) and increased MCA PI predicts incident dementia (Chung et al., 2017). In this study, the group difference in MCA PI was attenuated when adjusting for hypertension, consistent with the known bidirectional relation between hypertension and arterial stiffness (Nowak et al., 2018).

Impaired cerebrovascular reactivity to hypercapnia in patients with anemia secondary to kidney failure has been observed using positron emission tomography, as compared to healthy controls (Kuwabara et al., 2002). In contrast, in a recent study in a mouse model of CKD (DB1/2J mice with uremic vascular calcification), cerebrovascular reactivity to hypercapnia did not differ as compared to wild‐type mice, although the increase in blood flow was more rapid and decreased prematurely (Choi et al., 2021). This is consistent with a recent pilot study that reported impaired cerebrovascular reactivity to hypercapnia in chronic dialysis patients but not patients with non‐dialysis dependent stage 4–5 CKD, as compared to healthy controls (Slessarev et al., 2021). Cerebrovascular reactivity has been widely used as an index of cerebrovascular function and health, and predicts adverse clinical outcomes such as stroke, dementia, and mortality (Markus & Cullinane, 2001; Portegies et al., 2014; Wolters et al., 2016). However, a very recent publication supports that cerebrovascular reactivity to hypercapnia is only partially NO dependent, and NO is not obligatory for cerebrovascular reactivity to CO_2_ in young healthy adults (Hoiland et al., 2022). Thus, despite reduced NO bioavailability in CKD (Baylis, 2008), other compensatory vasodilators may be produced during hypercapnia, highlighting the critical importance of maintaining CBF (Ashby & Mack, 2021). In the present study, we utilized acetylcholine‐stimulated NO production in response to serum ‘exposure’ as a model of ex vivo endothelial cell function. The purpose of this model was to isolate the role of circulating factors in modulating cerebrovascular endothelial cell health and function, in general, rather than to assess NO as a specific mechanism of group differences in cerebrovascular reactivity. Interestingly, our serum incubation cell culture studies indicated a similar responsiveness of HBECs to acetylcholine stimulation after incubation with serum from patients with CKD as compared to healthy controls, suggesting that circulating factors in serum may not contribute to the reduced NO bioavailability previously described with CKD. Assessment of the role of circulating factors in modulating ex vivo endothelial function in endothelial cells from other vascular beds would help to confirm whether the lack of a group difference in NO bioavailability is specific to cerebrovascular endothelial cells. Additionally, these findings highlight preserved sensitivity to CO_2_ in particular in the setting of CKD.

Total CBF, assessed by ultrasonography of the internal carotid and vertebral arteries, was not significantly different in the CKD as compared to healthy control group, although notably higher in four participants with CKD (without obvious differences in other characteristics). Prior research on the influence of kidney function on CBF is unclear, with population‐based studies observing an association of lower eGFR with both reduced (Sedaghat et al., 2016) and increased (Tamura et al., 2016) CBF. Increased CBF may indicate impaired autoregulation, as CBF increases linearly with increasing blood pressure with loss of autoregulatory capacity (Tamura et al., 2016). Alternative explanations include increased metabolic demand, collateral circulation development, or anemia causing reduced arterial oxygen content (Tamura et al., 2016).

In the current study, participants with CKD exhibited a slower trail making part B time as compared to healthy controls, indicating impaired executive function. However, group differences were not observed in the NIH toolbox, limiting our interpretation of these results. Two‐thirds of the brain is supplied by the MCA, including areas that are involved with cognitive function (Harris et al., 2018). In addition to the association of MCA PI with trail making part B time, although NIH toolbox performance did not differ between groups, hemoglobin level was associated with NIH toolbox processing speed, which persisted after adjustment for age, sex, eGFR, and education. Interestingly, correction of anemia improves cognitive function, as well as resting MCA blood flow, in chronic hemodialysis patients (Shaker et al., 2018).

Patients with CKD had elevated circulating sCD14 levels as compared to healthy controls, which is a marker of gut‐blood barrier permeability. Elevated sCD14 has been described previously in chronic hemodialysis patients and correlates with levels of the gut‐microbiota metabolite trimethylamine N‐oxide (Hernandez et al., 2022). Disruption of the gut flora and intestinal barrier damage may promote translocation of uremic toxins and dysfunction in other organs (Lau et al., 2018). Consistent with this concept, higher levels of sCD14 were associated with higher MCA PI, after adjustment for age, sex, and eGFR. In contrast, no significant group difference was observed in levels of circulating BDNF or enolase 2, markers of blood–brain barrier permeability and disruption. Patients with CKD also had higher SOD activity in serum as compared to healthy controls, which is likely a compensatory mechanism to counter high production of reactive oxygen species characteristic of CKD (Akiyama et al., 2005).

To extend our findings to individuals with mildly impaired renal function and validate our findings from the primary cohort, we utilized a secondary cohort with cerebrovascular function measurements performed at a separate institution. Of note, this validation cohort consisted of generally healthy midlife and older adults with eGFR in the mildly decreased to normal range that were otherwise well matched for confounding variables (e.g., SBP, BMI). In accordance with results from our primary cohort, MCA PI was higher, but cerebrovascular reactivity to hypercapnia was not different, in adults with eGFR <86 ml/min/1.73m^2^ (group median) vs. those with eGFR ≥86 ml/min/1.73 m^2^. Furthermore, higher MCA PI was associated with lower eGFR, higher CFPWV, and slower performance on the Trails B test. Combined, these observations strengthen the primary findings that impaired kidney function, as defined by eGFR, is associated with greater cerebrovascular and aortic stiffness. That similar results were obtained in both cohorts despite slight variations in data collection (e.g., ventilatory rate, length of measurements) between the two institutions further validates the primary findings and provides confidence in the results.

There are several limitations to this study. Most notably, our sample size was small. However, we performed comprehensive physiological assessments and cognitive testing, and we were appropriately powered for our a priori primary outcome of cerebrovascular reactivity. An additional limitation is that group differences between the CKD and healthy control group other than the primary disease process, such as differences in BMI or the presence of diabetes and/or hypertension may have accounted for the observed results rather than CKD per se. However, such comorbidities are very common in patients with CKD, thus may contribute to phenotypic changes observed in patients with CKD, and even if beyond reductions in eGFR alone, are critical to the clinical presentation of this patient population. Hypertension and CKD (decline in kidney function) have a bidirectional and complex relationship (Hamrahian & Falkner, 2017). Additionally, patients with CKD were more likely to be prescribed antihypertensive agents; however, such medications would be expected to lower rather than increase pulsatility index (Webb, 2019). That similar findings were observed in our validation cohort that was matched for blood pressure, BMI, and medications suggests an underlying relation between kidney dysfunction and the cerebrovasculature.

Furthermore, due to budgetary constraints for necessary equipment, we were unable to assess beat‐to‐beat blood pressure in the primary cohort. Last, providing fixed inspired CO_2_ and measuring ETCO_2_ does not allow for standardization of the partial pressure of arterial CO_2_ (PaCO_2_) stimulus, but ETCO_2_ was the best noninvasive index of PaCO_2_ available to us, particularly given that arterial sampling is contraindicated in CKD patients who need to preserve the vasculature for potential vascular access for dialysis. The major strength of our study is that to our knowledge, this is the first study to assess MCA PI in patients with non‐dialysis dependent CKD, as well as assess cerebrovascular reactivity in early‐stage patients with CKD. Moreover, we also confirmed our main results in a validation cohort of generally healthy midlife and older adults with varied eGFR, further supporting the validity of our findings.

In conclusion, we have provided evidence that MCA PI is higher, but cerebrovascular reactivity is preserved in non‐dialysis dependent CKD. Further research is needed to confirm these results in larger cohorts and to delineate the mechanisms linking arterial stiffness, cerebrovascular function, and cognitive impairment in patients with CKD.

## AUTHOR CONTRIBUTIONS

E.O., K.A.F., D.H.C., and K.L.N were involved in conceptualization and writing—original draft. D.H.C. and K.L.N were involved in methodology. M.E.C. and K.L.N. were involved in validation and data curation. K.L.N. was involved in formal analysis and visualization. E.S.O., K.A.F., C.N.S., W.W., H.F‐B., M.J.R., D.H.C., and K.L.N were involved in investigation. D.R.S. and M.C. were involved in resources. C.N.S., W.W., H.F‐B., M.E.C., D.R.S., M.C., and M.J.R. were involved in writing—review and editing. D.R.S., M.C., D.H.C., and K.L.N. were involved in supervision. D.R.S., M.C., M.J.R., D.H.C., and K.L.N were involved in funding acquisition.

## CONFLICT OF INTEREST

None.

## ETHICS STATEMENT

All procedures were approved by the Institutional Review Board of the University of Colorado Anschutz Medical Campus and the University of Colorado Boulder campus and adhere to the *Declaration of Helsinki*. The nature, benefits and risks of the study were explained to the volunteers and their written informed consent was obtained prior to participation.
